# Cost-effectiveness of early detection of breast cancer in Catalonia (Spain)

**DOI:** 10.1186/1471-2407-11-192

**Published:** 2011-05-23

**Authors:** Misericordia Carles, Ester Vilaprinyo, Francesc Cots, Aleix Gregori, Roger Pla, Rubén Román, Maria Sala, Francesc Macià, Xavier Castells, Montserrat Rue

**Affiliations:** 1 Economics Department, Rovira i Virgili University, Reus, Catalonia, Spain; 2 Evaluation and Clinical Epidemiology Department, Parc de Salut Mar and CIBER of Epidemiology and Public Health (CIBERESP), Barcelona, Catalonia, Spain; 3 Terres de l'Ebre Region, Catalan Institute of Health, Catalonia, Spain; 4 Basic Medical Sciences Department, Biomedical Research Institut of Lleida (IRBLLEIDA)-University of Lleida, Lleida, Catalonia, Spain

## Abstract

**Background:**

Breast cancer (BC) causes more deaths than any other cancer among women in Catalonia. Early detection has contributed to the observed decline in BC mortality. However, there is debate on the optimal screening strategy. We performed an economic evaluation of 20 screening strategies taking into account the cost over time of screening and subsequent medical costs, including diagnostic confirmation, initial treatment, follow-up and advanced care.

**Methods:**

We used a probabilistic model to estimate the effect and costs over time of each scenario. The effect was measured as years of life (YL), quality-adjusted life years (QALY), and lives extended (LE). Costs of screening and treatment were obtained from the Early Detection Program and hospital databases of the IMAS-Hospital del Mar in Barcelona. The incremental cost-effectiveness ratio (ICER) was used to compare the relative costs and outcomes of different scenarios.

**Results:**

Strategies that start at ages 40 or 45 and end at 69 predominate when the effect is measured as YL or QALYs. Biennial strategies 50-69, 45-69 or annual 45-69, 40-69 and 40-74 were selected as cost-effective for both effect measures (YL or QALYs). The ICER increases considerably when moving from biennial to annual scenarios. Moving from no screening to biennial 50-69 years represented an ICER of 4,469€ per QALY.

**Conclusions:**

A reduced number of screening strategies have been selected for consideration by researchers, decision makers and policy planners. Mathematical models are useful to assess the impact and costs of BC screening in a specific geographical area.

## Background

In Catalonia (Spain), as in the majority of Western countries, breast cancer (BC) is the cancer with the highest incidence among women (almost 1/3 of all malignant neoplasms). BC causes more deaths than any other cancer (18% of the cancer deaths in Catalan women in the 1998-2002 period) [1]. Early detection and adjuvant treatments have contributed to the observed decline in BC mortality since the 1990s. However, there is debate over the optimal screening strategy, including frequency and starting and ending ages.

The economic cost of BC is also important. In the USA, BC drugs are the second biggest category of all pharmaceutical sales, growing at double the overall market [2]. Early detection of BC produces a stage shift in the direction of more favorable prognosis relative to the distribution of stages seen with symptomatic detection and may result in treatment savings. Nevertheless, early detection also affects the costs of initial treatment and follow-up. A new hypothesis has been proposed in recent studies, that improving the stage at diagnosis by early detection is unlikely to result in large treatment savings. The savings in treatment and palliative care for advanced BC may be counterbalanced by the high costs of more aggressive initial treatment and longer follow-up [3,4]. Since population based screening programs are funded by community resources, an economic evaluation that considers the long-term consequences of different scenarios both on outcomes (benefits and harms) and costs should be performed.

Economic evaluation is based on a comprehensive assessment of effects and costs. Cost-effectiveness analysis makes it possible to determine the cost of obtaining an additional unit of health outcome, comparing the costs and outcomes of different health interventions or strategies within the same intervention. Costs are valued in monetary units and effectiveness is valued in years of life, mortality reduction or quality adjusted life years (QALYs). What society is willing to pay for an additional unit of outcome is an open discussion. Most of the economic evaluation guidelines refer to this issue and propose different solutions. In Spain, Sacristan *et al. *proposed the amount of 30,000 € per QALY as a value limit for implementing a new strategy [5]. Other authors prefer to establish categories defined as feasible, possible or unfeasible, unless they are necessary to achieve a high-priority health policy goal.

In Spain, population based screening programs were established during the 1990s. Some economic evaluations of these programs have been performed. For instance, in Catalonia, Plans *et al. *[6] assessed the cost per woman screened and per cancer detected in 1996 and Beemsterboer *et al. *[7] used the MISCAN (Mlcrosimulation SCreening ANalysis) model to estimate the cost-effectiveness of early detection in 1998. In Navarra, Garuz *et al. *used decision trees to perform a cost-effectiveness analysis of the Navarra program and a marginal analysis to assess the inclusion of women 45-49 years old in the program [8]. Van den Akker-van Marle *et al. *used the MISCAN model to evaluate a pilot project in Navarra and to derive predictions of the long-run costs and effects of the program [9].

There are two major elements of the cost-effectiveness of mammography screenings that characterize a specific screening strategy: 1) the age interval and 2) the periodicity of the exams. In Spain, most of population-based early detection programs target women aged 50-69 and perform biennial mammograms. Extending the program to older or younger women has been a matter of interest for health policy-makers. In a previous work we analyzed the benefit, in terms of mortality reduction and years of life gained, of different screening strategies in Catalonia [10] using the Lee and Zelen stochastic models [11,12]. In the present work our goal was to perform an economic evaluation of these screening strategies, taking into account the cost over time of screening exams and subsequent medical costs, including diagnostic confirmation, initial treatment, follow-up and advanced care.

## Methods

Based on different BC screening recommendations, we generated 20 possible screening strategies by varying the periodicity of screening exams and the age intervals of women screened. We combined annual or biennial screening with age intervals that started at 40, 45 and 50 years and ended at 69, 70, 74 and 79 years. We also included the *background *or non-screening scenario. We used a probabilistic model to estimate the effect and costs over time of each screening scenario. We used the software Mathematica 7.0.1 [13], R 2.11.1 [14] and Stata 11.0 [15] for modeling and data analysis.

### The Lee and Zelen stochastic model

Lee and Zelen (LZ) developed a probabilistic model that predicts mortality as a function of the early detection modality. The characteristics and assumptions of the LZ model are described in detail elsewhere [11,12,16-19]. The assumptions of the LZ model are (1) a four-state progressive disease in which a subject may be in a disease-free state (S_0_), preclinical disease state (S_p_: capable of being diagnosed by a special exam), clinical state (S_c_: diagnosis by symptomatic detection), and a death from BC state (S_d_^bc^); (2) age-dependent transitions into the different states; (3) age-dependent examination sensitivity; (4) age-dependent sojourn times in each state; and (5) exam-diagnosed cases have a stage-shift in the direction of more favorable prognosis relative to the distribution of stages in symptomatic detection.

The basic LZ model calculates the cumulative probability of death for the cohort ν exposed to any screening program after *T *years of follow-up. Similarly, the cumulative probability of death for the cohort group without screening can be calculated. These probabilities were used to calculate the possible reduction in mortality from an early detection program after *T *years of follow-up. We extended the model to estimate incidence and prevalence. The inputs needed to model the Catalan data have been published elsewhere [20-22].

### Estimation of BC incidence, prevalence and mortality under different screening scenarios

We estimated the probability of being an incident case at age *u *(40 ≤ u < 80) and the probabilities of being a prevalent case or dying, each year afterwards, conditional to being diagnosed at age *u *(40 ≤ u < 80). These values were used to estimate the costs of initial treatment, follow-up and advanced care, respectively.

#### Incidence

Under a screening scenario, the probability of having a BC diagnosis at age *u*, was estimated as the sum of the probabilities of being detected at the screening exam and being diagnosed at the interval between two exams. The model uses *q(t)*, the probability density function of the sojourn time in pre-clinical state, S_p_. A detailed specification of the incidence estimation can be found in the Appendix in Additional file 1, sections C.1 and D.1.

#### Prevalence

Once a woman is diagnosed of BC, she can die from BC or from other causes. The proportion of women that remain alive at the end of the first year, *Prev(u*+1*)*, can be estimated as:(1)

where *I(u) *is the probability of being incident (symptomatic, detected by exam or interval case) in the interval [*u,u*+1), *m*^*-bc*^*(u) *is the central rate of death from other causes than BC in the same interval, and *D(*1*,u) *is the probability of death from BC during the first year after diagnosis at age *u *(Appendix in Additional file 1, sections C.3 and D.3).

The last summand of equation (1) represents the proportion of incident women that die from other causes in the age interval [*u,u+1*). We have assumed independence of BC and other causes of death and uniformity in the distribution of BC deaths in the age interval.

Similarly, for successive years *k*:(2)

#### Breast cancer mortality

Detailed equations for estimating the probability of death from BC at different time periods for different screening scenarios can be found elsewhere [10,12] and in the Appendix in Additional file 1, sections C.2 and D.2. Our model has used the BC survival functions from the US by age and stage for the period 1975-79 [22]. Since this period is prior to the introduction of mammography screening in Catalonia, the survival estimates were not affected by the lead time bias. Also, the US functions provided more favorable and realistic survival estimates than the Catalan ones because adjuvant treatments in Catalonia during the 1980s (pre-screening era) were less used than in the US.

#### Measuring the effect of different screening scenarios

For each screening scenario and for the background, we measured the effect of screening with three outcomes: 1) the number of lives extended (LE); 2) the number of years of life gained (YL); 3) the number of quality-adjusted life years gained (QALY). QALYs were estimated applying the weights derived from EuroQol EQ-5D quality-of-life utility scores that Stout *et al. *used in the US [23]. Quality-of-life weights for localized breast or regional cancer health states were assumed to be 90% or 75%, respectively, of the values for a healthy woman of the same 5-year age group, for a period of two years. Quality-of-life weights for the distant BC state were assumed to be 60% of the healthy state until her death. We also considered a loss of QALYs due to the anxiety derived from the screening mammogram itself (7 days at 25% of the healthy state) and from a false positive result (25 days at 25% of the healthy state). Stout *et al. *considered the loss of QALYs due to the test results' anxiety only in their sensitivity analysis.

All the calculations assumed an initial population of 100,000 women at birth. Incidence of BC and mortality from other causes refer to the cohorts born in the period 1948-1952. The time horizon for the study was 40-79 years of age.

### Costs' considerations

Costs can be categorized as direct (either healthcare or non-healthcare costs), indirect or intangible and each one of these categories is considered or not depending on the study's perspective and on the availability of data. We have adopted the perspective of the national health system and considered only the direct healthcare costs.

We have partitioned the estimation of costs into four parts: screening and diagnosis confirmation, initial treatment, follow-up and advanced care costs. All costs were valued in 2005 euros and both costs and outcomes have been discounted at an annual rate of 3%, according to the economic evaluation guidelines of the Spanish Ministry of Heath [24].

#### Costs of the breast cancer diagnosis under a screening scenario

The costs of screening mammograms, complementary tests and administrative expenses were obtained from the Early Detection Program of IMAS in the city of Barcelona. The program covers 42% of women living in Barcelona. We considered the following costs: screening mammogram plus administrative costs, 35 €; early recall mammogram, 23 €; non-invasive complementary tests, 298 €; and invasive tests, 473 €.

To obtain the costs of screening and diagnosis confirmation we made the following assumptions:

#### Part A): While women are screened

• All women at risk of BC in the target population participated in the screening exams and received a mammogram according to the periodicity and age interval of each screening scenario.

• There were 7% of women that received an additional mammogram for further assessment or early recall.

• We used the false positive (FP) rates for non-invasive and invasive tests obtained from a Spanish study that included eight Breast Cancer Early Detection Programs [25]. We multiplied the FP rates by the number of women at risk for BC to estimate the number of women that would receive additional non-invasive (e.g. ultrasound) or invasive tests (e.g. biopsy). See further details in Appendix E in Additional file 1.

• In the interval between exams there were no FP and all the women with BC diagnosed during the interval would undergo a non-invasive plus an invasive test.

• Sensitivity of mammogram was 0.55 for ages 40-45 years, 0.70 for 45-50 years, 0.75 for 50-70 years and 0.80 for > 70 years. These values, used previously by Lee and Zelen, were derived from the Breast Cancer Surveillance Consortium database which contains mammogram screening data and follow-up for approximately one million US women, dating back to 1994 [12].

The results obtained when applying the FP rates to the target population allowed us to estimate the ratio of negative results/positive results for invasive tests and the ratio of non-invasive/invasive tests (Table 1, columns 2 and 4). These ratios are different for each of the scenarios because they depend on the number of BC diagnosed. Also, these ratios are age-specific and decrease linearly with age after the sixth year from the start of the screening.

**Table 1:** Ratios Non-invasive/Invasive tests and Invasive test -/Invasive test + for a screening scenario with annual periodicity and exams in the age interval 50 to 69 and for the background scenario

Age	Non-invasive/invasive tests		Invasive test -/Invasive test +	
	
	Screening	Background	Screening	Background
**50**	6.92	2.54	2.03	0.57
**55**	6.19	2.32	0.65	0.5
**60**	4.21	2.1	0.42	0.42
**65**	3.79	1.89	0.35	0.35
**70**	1.67	0.28		
**75**	1.46	0.2		

#### Part B): After the last screening exam

For screening scenarios where the last screening exam was performed before the age of 79, we proceed as follows:

• Since we do not have FP rate estimates for a population without screening, we assumed that the ratio of benign to malignant biopsy was the same among screened and non-screened women [26]. We linearly projected the ratio for non-invasive/invasive tests during the screening interval up to age 79, for each screening schedule.

• We multiplied the projected ratios by the number of women diagnosed with BC (true positives) obtained from the LZ model and we obtained the number of FP for the invasive tests.

• We also linearly projected the ratio of non-invasive/invasive tests during the screening interval, up to age 79.

• We considered that the cumulative rate of invasive tests in screened women was double this rate in non-screened women [26].

• We multiplied the estimated non-invasive/invasive ratio by the number of invasive tests to obtain the number of non-invasive tests.

#### Costs of the BC diagnosis under the background scenario

For the background scenario, the cost for non-invasive and invasive complementary tests are the same as for screening. The cost of mammogram for background was the same as for early recall screening mammography, 23 €.

To obtain the number of non-invasive and invasive tests and the FP rates under the background scenario, we proceeded as described in *part B*) of the previous section. However, under the screening scenario the ratios of negative/positive results for invasive tests and non-invasive/invasive tests were higher for the six initial exams and then decreased linearly. Therefore, we excluded the first six ratios and projected the linear section of the series obtained from a population with annual periodicity of exams in the age interval 50-69 years (Table 1, columns 3 and 5. Also, see Appendix E in Additional file 1).

#### Costs of the initial treatment, follow-up and advanced care

Data on costs was obtained from a database that included 592 women consecutively diagnosed and initially treated for BC at the IMAS-Hospital del Mar in Barcelona in the period January 1st, 2000 - December 31, 2003. Cost categories are shown in Table 2. There were 68 (11.5%) *in situ *cases which were excluded from the analysis. The distribution of disease stages at diagnosis of invasive tumors was 196 (42%) Stage I, 163 (35%) Stage II, 74 (16%) Stage III, and 32 (7%) Stage IV.

**Table 2:** Costs' categories

Cost category	Items
**In-hospital**	Length of stay by specialty ward
**Ambulatory visits**	Type of ambulatory visit
	Emergency visits
**Chemotherapy**	Drugs
	Treatment protocol
**Other hospital costs**	Other drugs
	Lab tests
	Radiological tests
**Radiotherapy**	Number and type of courses
**Hormone therapy**	Drugs

**Initial treatment costs **were higher when the BC was diagnosed in a more advanced stage. The initial treatment lasted approximately one year. The mean costs for non-metastatic disease were 9,529 € for Stage I, 14,184 € for Stage II and 16,898 € for Stage III. To the previous values we added 638 €, the cost of adjuvant tamoxifen for five years, which was prescribed to 67.6% of women diagnosed with a Stage I, II and III. For women diagnosed at stage IV, the initial treatment cost was assumed to be zero and we considered only diagnosis and advanced care costs.

**Follow-up costs **for women diagnosed with BC include ambulatory visits and diagnostic tests that women receive, starting the year after the BC diagnosis. Based on the data, we assumed that the cost during the first year of follow-up was 1,365 € and 530 € for the following four years.

**Advanced care cost **was obtained using data from the 32 patients diagnosed with metastatic cancer, followed during five years. Survival at the end of the period was 37.5%. We estimated the cost of advanced care as the mean of all the direct costs from diagnosis until death or last follow-up, 28,413 €. Costs for living women after five years of follow-up did not change the mean significantly.

#### Cost-effectiveness analysis

To compare the relative costs and outcomes of the different scenarios, we calculated the incremental cost-effectiveness ratio (ICER). The ICER is defined as the ratio of the change in costs to the change in effects of a specific intervention compared to an alternative. The ICER indicates the additional cost of obtaining one additional unit of outcome. We have compared each scenario with the next most effective alternative. Strategies were classified into three categories: non-dominated, dominated and extended dominated. A strategy was considered *dominated *if it was more expensive and less effective than another. Extended dominance occurs when a strategy is improved by mixing with two other strategies. When costs are plotted on the *y *axis and outcomes on the *x *axis, a dominated strategy lies above and to the left of the non-dominated strategy.

Once dominated or extended dominated strategies are excluded, the remaining strategies form the *cost-effectiveness frontier*, the *efficient *alternatives for which no alternative policy exists that result in better effects for lower costs. Usually there is a social threshold, or willingness to pay, that constrains the choice between efficient strategies. Finally, a rational decision maker has to decide whether or not to move up the efficiency frontier.

#### Sensitivity analysis

We performed a sensitivity analysis to study the impact on our conclusions when some of the inputs were modified. First, we investigated the effect of increasing all costs that could be due to, for instance, the introduction of new drugs. Second, we examined the impact of longer follow-up times. Third, we changed the ratio of screening/background non-invasive tests because there is limited information for estimating this ratio with high confidence. Fourth, we set the screening participation to 50% in the screening program. Fifth, we doubled the cost of invasive tests for screen-detected tumors to account for the difficulty of detecting non-palpable lesions. All the tested scenarios can be found in Table 3. For this analysis we present LE and QALYs as measures of the effect (the results obtained with YL and QALYs were similar).

**Table 3:** Baseline assumptions and ranges tested in the sensitivity analysis

Parameter	Baseline model	Sensitivity analysis
**Initial treatment cost by stage**	I: 9,960 €, II: 14,616 €, III: 17,329 €	2, 3, 4, 5, 10-fold
**Follow-up cost**	1,365 € 1st yr., 530 € afterwards	2, 3, 4, 5, 10-fold
**Advanced care cost**	28,413 €	2, 3, 4, 5, 10-fold
**Years of follow-up**	5	11, 16, 21
**Screening/background noninvasive test**	2	1, 3
**Screening participation**	100%	50%
**Screening/background cost of invasive tests**	1	2

## Results

**Figure 1:**
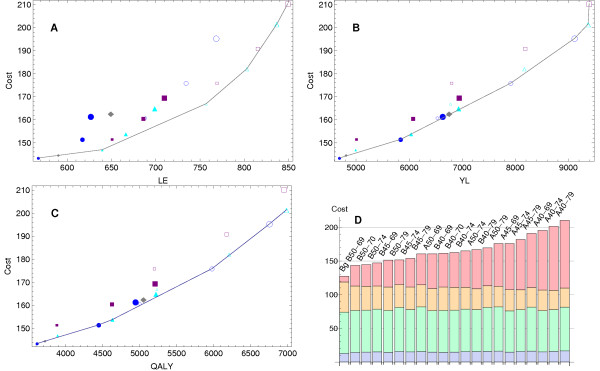
**Cost and effectiveness**. Cost-effectiveness analysis of different screening strategies: **A - **Incremental cost (×10^6 ^€) per life extended (LE); **B - **per year of life (YL); and **C - **per quality-adjusted life year (QALY). Empty figures correspond to annual strategies and full figures to biennial. Screening start age: 40 (big), 45 (medium) and 50 (small). Screening end age: 69 (circle), 70 (diamond), 74 (triangle), and 79 (square). The line joins the dominant scenarios. **D - **Cost for all strategies by phase: detection (pink), initial treatment (green), follow-up (blue), and advanced care (yellow).

Figures 1A, 1B and 1C present the results of the cost-effectiveness analysis when the effect measures are LE or YL or QALYs, respectively. The background scenario was the reference scenario. All the screening alternatives represented increased effectiveness and costs with respect to the background scenario.

Table 4 shows the non-dominated or non-extended dominated alternatives for each effectiveness measure. The ICER indicates the cost of obtaining an additional unit of effect when moving from one strategy to the next.

**Table 4:** Cost-effectiveness of mammography screening strategies in Catalonia (Spain)

Scenario	Cost	ΔCost	Effect	ΔEffect	ΔC/ΔE (ICER)
			
	**(×10**^**6 **^**€)**	**(×10**^**6 **^**€)**	LE	ΔLE	€/LE
**Bg**	127.3		0		
**B 50-69**	143.4	16.2	567	567	28,465
**B 50-70**	144.6	1.1	590	23	49,184
**B 50-74**	147.0	2.5	640	50	50,188
**A 50-74**	167.0	20.0	757	117	170,304
**A 45-74**	182.1	15.1	803	46	330,098
**A 40-74**	201.5	19.4	837	34	573,062
**A 40-79**	210.4	8.9	849	12	715,941

			**YL**	**ΔYL**	**€/YL**

**Bg**	127.3		0		
**B 50-69**	143.4	16.2	4,691	4,691	3,444
**B 45-69**	151.5	8.1	5,842	1,151	7,015
**A 45-69**	176.0	24.4	7,917	2,075	11,777
**A 40-69**	195.4	19.4	9,117	1,200	16,166
**A 40-74**	201.5	6.2	9,370	253	24,415
**A 40-79**	210.4	8.9	9,390	20	451,370

			**QALY**	**ΔQALY**	**€/QALY**

**Bg**	127.3		0		
**B 50-69**	143.4	16.2	3,614	3,614	4,469
**B 45-69**	151.5	8.1	4,447	833	9,694
**B 45-74**	153.9	2.4	4,633	186	12,633
**A 45-69**	176.0	22.1	5,979	1,346	16,411
**A 40-69**	195.4	19.4	6,756	777	24,975
**A 40-74**	201.5	6.2	6,987	231	26,720

Incremental cost per effect (LE, YL and QALY) assuming a cohort of 100,000 women at birth. Dominated or extended-dominated strategies are not included.

The detailed results for all the 21 scenarios can be found in Table A.1 in Additional file 1.

### Measuring effectiveness with number of lives extended (LE)

Figure 1A and Table 4, upper section, present the results of this analysis. Among the 20 studied screening strategies and the background scenario, 13 were either dominated or extended dominated by other strategies. Three of the seven screening scenarios that should be taken into consideration were biennial and four annual: B50-69, B50-74, B50-70, A50-74, A45-74, A40-74 and A40-79. Only one of them had the last exam at age 79, two scenarios started at age 40, one at age 45 and four at age 50.

Moving from the background to B50-69, the current public screening program in Catalonia, represented an incremental cost of 28,465 € per LE. Moving from B50-69 to the next alternative, B50-70, represented an incremental cost of 49,184 € per LE. The ICER increased considerably when moving from biennial strategies to annual strategies. The last non-dominated strategy, A40-79 had an ICER of 715,941 €, which should be interpreted with caution because we did not account for the reduction in mortality after age 79.

### Measuring effectiveness with years of life gained (YL)

Figure 1B and Table 4, middle section, show the results of this analysis. Among the selected strategies there were six scenarios, two of them biennial and four annual: B50-69, B45-69, A45-69, A40-69, A40-74 and A40-79. Three of the scenarios, B50-69, A40-74 and A40-79, were also selected when the effect was measured as LE.

All the selected alternatives, except A40-79, had an incremental cost lower than 30,000 € per additional YL and therefore, could be implemented if economic resources were available. As for the LE analysis, the results for alternative A40-79 should be interpreted with caution.

### Measuring effectiveness with quality-adjusted life years (QALYs)

Figure 1C and Table 4, lower section, show the results of this analysis. Six screening scenarios, three biennial and three annual, were selected: B50-69, B45-69, B45-74, A45-69, A40-69 and A40-74. Five of these scenarios were also selected when the effect was measured as YL and two of them when the effect was measured as LE. Compared with the current public screening strategy, B50-69, all the remaining selected scenarios started the exams earlier and two of them ended later. All the selected alternatives had an incremental cost per QALY lower than 30,000 € and therefore could be considered for implementation.

### Sensitivity analysis

Tables A.2, A.3 and A4 in Additional file 1 show the selected scenarios after modifying costs, length of follow-up and the ratio of non-invasive tests among screened and background strategies. Overall, the sensitivity analysis showed that conclusions were not affected by small or moderate changes in the inputs of the model.

The input that caused more changes in the selected screening scenarios was the advanced care cost. When advanced care cost was three times higher than the initial value, the background scenario was no longer the least expensive scenario. The following scenarios: B50-74, B50-70 and B50-69, in that order, had lower costs than the background. At the extreme value of advanced care cost (10-fold) only annual screening strategies were selected.

When we changed the follow-up time from four to ten or twenty years, the selected scenarios were the same as the baseline except for B50-70, which became dominated when the effect was measured in LE. The selected screening scenarios did not change after modifying the ratio of non-invasive tests among screened and background strategies. Assuming that only 50% of the invited population is screened produced only slight changes in the selected scenarios but increased the ICER considerably. When costs of invasive tests were doubled for the screened women there were no changes in the selected scenarios, there were only slight changes in the ICER values.

## Discussion

### Principal findings

This study performed an economic evaluation of different BC mammography screening strategies in Catalonia (Spain), using mathematical models. We assumed the perspective of the national health system and considered the direct healthcare costs over time of screening, diagnosis, initial treatment, follow-up and advanced care.

Our results show that, based on the incremental cost-effectiveness ratio (ICER), a reduced number of strategies can be selected for consideration from a set of 20 screening scenarios. Strategies that start at age 50 and end at age 74 predominate among those selected when the effectiveness of screening is measured in terms of the number of lives extended, and strategies that start at the ages 40 or 45 and end at age 69 predominate when the effect is measured as YL or QALYs. Independently of how the effect is measured, the ICER increases considerably when moving from biennial to annual scenarios.

An interesting result is that, assuming 100% participation in the studied screening strategies, the background is always the reference scenario because it has the lowest cost. But the effectiveness of the background is the lowest, for all the effect measures. In addition, once the non dominated scenarios are ordered according the ICER, always the next alternative is B50-69 that corresponds to the current public screening program in Catalonia. However, in some cases, other alternatives are more effective at a cost that could be considered implementable, given the generally accepted reference values. When the effect is measured as YL or QALY, these alternatives start the screening at ages younger than 50 years whereas only a few suggest to finish after the age 69.

Sensitivity analysis showed that the results were robust to moderate changes in costs of treatment or length of follow-up after initial treatment. Only dramatic increases of advanced cancer care modified the scenarios selected in the cost-effectiveness analysis in favor of annual screening scenarios. We did not perform a sensitivity analysis of changes in the survival functions as a result of improvements in mortality and prognosis after a BC diagnosis. The CISNET groups verified that there was a negative interaction between screening and adjuvant treatments. That means that the benefits of screening are smaller if treatments are more effective. As Cronin *et al.* pointed out, taken to the extreme, if treatment were completely curative there would be no additional mortality benefit associated with early detection [27].

### Costs of diagnosing and treating breast cancer

Many authors have studied the costs of diagnosing and/or treating BC [3,4,28-31]. There is high variability in the methodology, patient characteristics, perspective and time horizon used. Some authors calculate the net costs by subtracting the costs of care of age-matched controls [28,30]. Other authors identify the cancer-associated costs, which requires someone to decide which costs should be included. Some studies restrict the analysis to pre- or post-menopausal status [4] or to screened or non-screened groups. The objectives of studying costs also can be very different, from performing a descriptive study of costs from diagnosis to death [29] to building a simulation model [31]. Nevertheless some characteristics are common to most of the studies, for instance, the acceptance of increasing costs over time of advanced cancer care, and the substantial weight of hospitalization costs [28,29,31].

A major challenge is to estimate the costs of advanced disease. Even though clinical practice guidelines provide standard treatment for advanced disease, very often treatments are customized according to the tumor or the patient's characteristics and the response to each treatment line. De Koning *et al. *[32] and Richards *et al. *[33] pioneered the economic evaluation of advanced cancer care in the early 1990s when screening programs were spreading and information about their impact was needed. Recently, Guest *et al. *estimated the costs of palliative care for BC as £2,482 (at 2000/2001 prices) per patient [34]. Berkowitz *et al. *[35] assessed the lifetime direct costs of treating metastatic disease using the Statistics Canada's Population Health Model. On average, women with metastatic disease were expected to live three years and to incur direct treatment costs of approximately $60,000 per case, in 1998 US dollars. In comparison, patients treated for metastatic BC in our study had, on average, lower costs, 28,413 €. These differences may be explained, in part, by financial and organizational differences in health systems. In 2005, health care expenditure measured in PPP (Purchasing Power Parity) per capita was $2,225 in Spain, $3,326 in Canada and $6,401 in the USA [36].

Campbell *et al. *reviewed 29 cost-of-illness studies for BC in the US [37]. Of these, 22 measured only direct medical costs and took the health payer perspective. The estimated lifetime per-patient costs ranged from $US 20,000 to $US 100,000. The costs of initial and terminal treatments were greater than follow-up care on a per-unit-time basis, but follow-up care accounted for the largest part of lifetime cost due to the relatively long survival of BC patients. In our study, the estimated cost over time per patient fluctuated between 26,000 and 35,000 € depending on the intensity of early detection exams (no screening or annual screening in the 40-79 years of age interval, respectively). When a 3% discount was applied, cost over time per patient oscillated between 25,600 and 37,000 € (Table A.1 in Additional file 1).

The distribution of costs by phase in our study is consistent with the results found in the literature. When averaging the cost by phase over the different scenarios, for a cohort of 100,000 women at birth, the highest cost corresponded to initial treatment (63,083,670 €), followed by detection cost (55,353,560 €), advanced cancer care (33,011,915 €) and the costs of follow-up (3,031,631 €).

### Cost-effectiveness of mammography screening

Several authors have studied the cost-effectiveness of mammography screening using mathematical models [23,38-41]. Generally, these studies assess the effect of different screening strategies in relation to no screening. Some of them include the current guidelines or the actual screening scenarios among the compared screening strategies.

Wong *et al. *studied the cost-effectiveness of mammography screening in Chinese women in Hong Kong, adopting a societal perspective [39]. They compared biennial alternatives beginning at ages 40 or 50 and ending at ages 69 or 79, with the results from no screening. The least costly, non-dominated strategy was screening from ages 40 to 69 years, with an ICER of $61,600 per QALY saved or $64,400 per life year saved compared with no screening. These values were much higher than ours or other found in the literature. A difference with our study is that Wong *et al. *included ductal carcinoma in situ (DCIS) cases. Lee *et al. *[41] studied the cost-effectiveness of mammography screening in Korea using the model proposed by Lee and Zelen [12]. The effectiveness of mammography screening was defined as the probability of detecting BC in the preclinical state and the cost was based on the direct cost of mammography screening and confirmative tests. They concluded that biennial mammography screening for women aged at least 40 years was cost-effective. Ahern *et al. *assessed the cost-effectiveness of screening strategies recommended by the National Cancer Institute, the American Cancer Society (ACS), and the US Prevention Services Task Force in the USA and compared them with alternative strategies, using a microsimulation model. Mammography and clinical breast exams in alternating years from ages 40 to 79 years was a cost-effective alternative compared with the guidelines, costing $35,500 per QALY saved compared with no screening. The ACS guideline was the most effective and the most expensive, costing over $680,000 for an added QALY compared with the above alternative. The authors concluded that strategies with lower costs and benefits comparable with those currently recommended should be considered for implementation in practice and future guidelines.

In Spain, Plans *et al.*, in 1996, compared the direct health service costs of a round of screening for a program that screened women aged 50-64 years with the resulting estimated cancers detected [6]. They estimated a cost per women screened of $350 and a cost-effectiveness ratio of $7,020 per year of life gained. Garuz *et al.*, in 1997, performed a cost-effectiveness analysis of a BC mammography screening program that consisted of a biennial mammography in all women 50-64 years old [8]. They used data from the Navarre Screening Program, the Guipuzcoa Cancer Registry and the literature. Costs were estimated using a Markov model and measured in 1993 ECUs (1 ECU = 1 €) and a discount of 6%. The cost-effectiveness ratio per avoided death was 115,500 ECUs and 7,300 ECUs per saved life year. Extending the program to women 45-49 years represented an incremental cost of 229,000 and 9,400 ECUs, respectively. We did not analyze the strategy for ages 50-64, but our biennial 50-69 strategy, compared with the background, resulted in around 28,500 € per avoided death and 3,500 € per year of life saved. Extending the program to the 45-49 age group would represent an incremental cost of 162,000 and 7,000 €, respectively. Our values are lower than those reported by Garuz *et al. *This may be explained by differences in cost assessment, in the discount rate and time value of money (6% and future value in Garuz *et al. *versus 3% and present value in ours) and in the age at the last exam, 64 versus 69 years. The non-discounted cost per life saved in the Garuz *et al. *study was 38,400 €, a value more similar to ours.

Beemsterboer *et al. *[7] using Catalan data on BC mortality, incidence and screening and Dutch data on costs, obtained cost-effectiveness ratios equivalent to 5,553, 4,387 and 4,321 € per YL (5% discounting) for scenarios that target women 50-64 years of age with screening intervals of one, two and three years, respectively. De Koning *et al.*, in 2000, reported the cost per YL of screening with biennial mammograms as 2,650 € in Navarra (ages 45-65), 4,475 € in Catalonia and 7,125 € in Spain (both for ages 50-69) [42]. Our result, for B50-69, was 3,555 € per YL, compatible with the Beemsterboer and de Koning results, even though the costs of treating BC have increased in the last decade.

### Limitations

We have used a very detailed model that allowed us to thoroughly assess the cost and effectiveness of different screening scenarios. However, our study has several limitations, among them are the following. Our model relies on data and assumptions that may be not correct. When available, we have used Catalan or Spanish data from population based registries or BC screening programs. If the input data was not available at the region or country level, we used data that the CISNET had prepared for BC mortality modeling research groups in the USA [43]. Finally, there were some inputs that Lee and Zelen had obtained from published randomized clinical trials and observational studies. In a previous publication, we performed a sensitivity analysis to assess the effect of departures from the modeling assumptions on the effectiveness of early detection [10] and we concluded that the model was robust.

We have not obtained confidence intervals of the model outputs. Our model is probabilistic because it works with the probability density functions of the different inputs related to the natural history or detection of BC. It is also an analytic model that consists of a set of equations describing BC mortality over time. There is uncertainty associated with the model inputs and there is also uncertainty associated with the model structure. It is complex and computationally intensive to obtain the variance of the model estimates. Instead, we have carried out a sensitivity analysis to explore how changes in the input parameters affect the results.

With respect to the outcome measures, we have included LE as a measure of effect together with YLG and QALYs. We want to highlight that the standard and internationally recognized measure to compare different health interventions and measure their effectiveness is the QALY [44]. Although it can be interesting to estimate the amount of LE, this measure is less robust as an outcome measure and cannot be recommended as the basis for population-based policy decisions.

With respect to costs, we have several considerations. a) Costs were obtained from a single public hospital in Barcelona, which may not be representative of the hospitals in the region. However, we believe that the costs of diagnosing and treating BC in the hospitals of the Catalan public health system are not remarkably different. b) Advanced care costs were obtained from a small sample of metastatic BC patients at diagnosis. About one third of them were still alive after five years. Including living patients in the calculations may have underestimated the average cost of treating advanced disease. Excluding living patients from the analysis would have produced a biased sample. More adequate methods, based on the Kaplan-Meier sample average estimate [45], would have produced a better estimate, but we did not have all the necessary information to apply them, such as the costs of treatment over time on a monthly basis. c) Innovative treatments for advanced BC that currently may represent an improvement in survival or quality of life as well as an increase in cost were not available in the 2000-2003 time period. Despite these issues, sensitivity analyses showed that the results only changed when costs varied dramatically. d) We restricted the study to direct healthcare costs and the perspective of the national health system because the sources of indirect costs and the methods used to estimate them are heterogeneous, thus the estimates have high variability depending on the approach adopted. Reviewing some works that analyze the differences in the total costs (direct and indirect) for cancer patients in Spain, we found that indirect costs can represent from 20% to 70% of the total costs [46,47]. Obviously, the results that we present could have been very different if indirect costs had been considered.

The decision of implementing a specific alternative is influenced by the budget assigned to the screening program and also by the amount that society is willing to pay for each effectiveness unit. Since the number of LE or mortality reduction is not a standard effectiveness measure in economic assessments, is not easy to find reference values for comparison.

There is scarce information in the literature about rates of false positives for invasive and non-invasive tests to diagnose BC in a non-screened population. Again, the sensitivity analysis showed that the selected scenarios were robust to changes in the assumptions.

Our study did not take into account either overdiagnosis of BC as a consequence of screening or DCIS. The impact of overdiagnosis would be to increase costs and decrease quality of life. Strategies with a higher number of screening exams (annual) would have a higher incremental cost per additional effect unit and, therefore, would end up being dominated by less intensive screening strategies (biennial). We have not accounted for overdiagnosis because there is high variability in overdiagnosis estimates. When we studied overdiagnosis in our region, we obtained a high association between exposure to screening and an increase in incidence, beyond what was expected by the advance of diagnosis, but the precision of the overdiagnosis estimate was low [48]. We would like to have a more precise estimate of overdiagnosis before including it in the model.

In our model, DCIS cases were not included. The researchers that developed the probabilistic model that we have used considered that available information on the natural history of *in situ *disease was insufficient to include *in situ *tumors in the model [11]. When observing the results of the CISNET groups in the USA, there does not seem to be a pattern between the screening benefits (in terms of mortality reduction) produced by the models and whether or not they modeled *in situ *disease [27]. Nevertheless, including DCIS would increase the costs of treatment and decrease quality of life for the DCIS tumors that would not progress, similarly to overdiagnosis. We plan to incorporate both, DCIS and overdiagnosis, in future models.

## Conclusions

We have studied the cost-effectiveness of several BC screening scenarios and have selected a reduced number of them for consideration by researchers, decision makers and policy planners. Mathematical models have been useful to assess the impacts and costs of BC screening interventions, accounting for the population and epidemiological data of a specific geographical area.

## Competing interests

The authors declare that they have no competing interests.

## Authors' contributions

MC and MR codeveloped the project that includes this study, participated in the statistical analysis, wrote drafts and obtained author's feedback. MC, AG and FC developed costs estimations, performed the cost-effectiveness analysis and participated in writing and revising the manuscript. EV developed the computer programs that estimate incidence, prevalence, mortality, and costs, and participated in writing and revising the manuscript. RP codeveloped the project and participated in the interpretation of data. RR and FM provided data from the RAFP project and the hospital Cancer Registry, and participated in revising the manuscript. MS and XC coordinated the RAFP project and participated in revising the manuscript. All authors read and approved the final version of the manuscript.

## Pre-publication history

The pre-publication history for this paper can be accessed here:

http://www.biomedcentral.com/1471-2407/11/192/prepub

## Supplementary Material

Additional file 1 **Appendix**. The file contains further details of the model for the estimation of BC incidence, prevalence, mortality, false positive tests, and additional tables. Click here for file
