# Supplementary material for: Cost-effectiveness of early detection of breast cancer in Catalonia (Spain)
Source: BMC Cancer. 2011 May 23;11:192. doi: 10.1186/1471-2407-11-192 (PMC3125279; doi:10.1186/1471-2407-11-192)
Supplement: Additional file 1 — Appendix. The file contains further details of the model for the estimation of BC incidence, prevalence, mortality, false positive tests, and additional tables. [file 1471-2407-11-192-S1.PDF]

APPENDIX

A Introduction to the mathematical model

Data obtained under the no-screening scenario is compared to data obtained under the screening scenario, therefore some of the notation is common for both situations and follows the Lee and Zelen (LZ) work [1].

The age at which the study starts is denoted by z. In the screening scenario z is the age at which the first screening exam is performed and $t_0 = 0$ the time at which the first screening exam is performed or origin time.

There are three chronological times (relative to $t_0 = 0$) that must be taken into account in the formulas. These are: $x=$ time at which the preclinical state, S_p, is entered, (x can be positive or negative), $\tau=$ time at which the clinical state is entered, and $y=$ time at death. The corresponding ages are $z + x$, $z + \tau$ and $z + y$ and the sojourn times in S_p and S_c are $(\tau - x)$ and $(y - \tau)$, respectively.

We present the formulas used to estimate BC incidence, mortality and prevalence for a specific cohort ν. Survival and incidence probabilities and mammography sensitivity vary by age and cohort of birth. For simplicity, the index ν that indicates the birth cohort is not shown in the notation.

B Estimation of number of women at risk of breast cancer $S(t)$

The probability of surviving free of disease $S(t)$ is one of the inputs to the mathematical model. It depends on mortality from causes other than breast cancer (competing risks) and BC incidence. To estimate $S(t)$ we performed the following steps:

1. Obtain the central mortality rate at age u from other causes than breast cancer

$$m^{-bc}(u) = m^{\tau}(u) - m^{bc}(u) \tag{1}$$

   The all-cause and BC central death rates are labeled as m^{τ} and m^{bc}, respectively. The numerators of the central rates are the number of deaths in the age interval. The denominators have been approximated by the interval midpoint population.

2. Assume that the instantaneous force of mortality λ_m^{-bc} is constant over the age interval $[u, u+1)$ and can be approximated by $m^{-bc}(u)$.

3. Obtain the central incidence rate $i(u)$ and assume that it can approximate the instantaneous incidence rate λ_i. Breast cancer incidence was modeled with an age-cohort model [2].

4. Obtain the hazard of failure either due to being a BC case or dying from other causes (λ^F). We assumed that the two hazards were independent.

$$\lambda^F = \lambda_m^{-bc} + \lambda_i \tag{2}$$

5. Solve the differential equation:

$$S'(t) = \lambda^F S(t); \quad S(0) = 1 \tag{3}$$

Mortality data was obtained from the Catalan Mortality Registry and the National Institute of Statistics (INE) [3,4]. Overall mortality data was available for calendar years 1900-2004 and breast cancer mortality data was available for calendar years 1975-2004. Population estimates were obtained from the INE and the Catalan Statistics Institute (IDESCAT) [4,5].

Incidence data from the population-based cancer registries of the Catalan provinces Girona and Tarragona was used. These two registries cover 20% of the Catalan population. The available periods with information on BC incidence were 1980-1989 and 1994-2002 for Girona and 1983-1997 for Tarragona. We obtained the observed incidence rate by combining both sources of information and using the population counts of the official census for the same time periods [4].

C Background scenario

This section refers to the *background* scenario where BC is diagnosed by usual care with no screening.

C.1 BC incidence

The probability of being incident at the time interval $[t, t+1)$ was estimated using the formula:

$$I(t) = \int_t^{t+1} S(u)i(u)du \tag{4}$$

where $i(u)$ is the age and cohort specific incidence at time u [2].

The proportion of incident women by stage, j, was estimated using the distribution of disease stages at diagnosis for background cases π_{bg}:

$$I(t, j) = \pi_{bg}(t, j)I(t) \tag{5}$$

C.2 Probability of dying from BC

As stated in previous LZ's works [1, 6], the probability of dying from BC T years after the start of the study, in the absence of screening, can be estimated using the formula:

$$D(T) = \int_T^{T+1} \int_0^y \Phi(y-\tau)S(z+\tau)i(z+\tau)g_{bg}(y-\tau)d\tau dy \tag{6}$$

where g_{bg} is the probability density function (pdf) of surviving obtained by weighting the age and stage-specific pdfs by the stage distribution for background cases π_{bg} [1, 6]

$$\Phi(t) = \begin{cases} 1 & t \geq 0 \\ 0 & otherwise \end{cases} \tag{7}$$

C.3 Prevalence of BC

Prevalence of BC depends on incidence, death from BC and other causes. We defined $Prev(u+1)$ as the proportion of women that remain alive at the end of the first year after diagnosis, see Equation (1) in Methods.Then $D(1, u)$ is the probability of death from BC during the first year after diagnosis at age u and can be estimated as:

$$D(1, u) = \int_0^1 \int_u^{u+1} \phi(y-\tau)S(z+\tau)i(z+\tau)g_{bg}(y-\tau)d\tau dy \tag{8}$$

Similarly, for successive years k, Prev(u+k) is the proportion of women that were incident at age u and remain alive at the end of the year k, see Equation (2) in Methods,where $D(k, u)$ is estimated as:

$$D(k,u) = \int_{u+k-1}^{u+k} \int_{u}^{u+1} \phi(y-\tau)S(z+\tau)i(z+\tau)g_{bg}(y-\tau)d\tau dy \quad (9)$$

We have computed $I(u)$, $D(k,u)$ and $Prev(u+k)$ for u in $40,...,79$ years of age and k in $1,...,79-u$.

D Screening scenarios

This section presents the formulas used to perform the estimations under a generic screening scenario. A pattern of screening is characterized by age interval and periodicity of exams. Survival distribution functions and stage at diagnosis distribution are pattern specific.

D.1 BC incidence under screening

D.1.1 Relation between BC incidence and transition to the pre-clinical and clinical states S_p and S_c

Under screening we do not observe the time at which a BC will become symptomatic. Therefore, the incidence distribution can not be used in the calculations. Instead, the LZ models use the transition probability $S_0 \rightarrow S_p$ denoted by $w(t)dt$ and the probability density function of the sojourn time in a preclinical state, $q(t)$ [7].

The following equation shows the equivalence between the estimation of incidence at age $z+k$, $k = 0, 1, 2...$ using the incidence $i(t)$ or the $w(t)$ and $q(t)$ functions.

$$I(z+k) = \int_{k}^{k+1} S(z+\tau)i(z+\tau)d\tau \approx \int_{k}^{k+1} \int_{0}^{z} S(\tau-x)w(\tau-x)q(x)dxd\tau \quad (10)$$

In the following sections we use this equivalence to estimate the probabilities under screening.

Under a screening scenario, the LZ models distinguish if BC was detected in a screening exam or if it was diagnosed in the interval between two screening exams.

D.1.2 BC detected at exam r

The LZ model considers n examinations given at chronological times $t_0 < t_1 < ... < t_{n-1}$. The first exam is performed at age z and time $t_0 = 0$. Each successive exam r is performed at time t_{r-1}. For instance:

Exam number (r)	1	2	3	...	r	...	n
Exam time for annual screening, in years (t_{r-1})	0	1	2	...	$r-1$	...	n-1
Exam time for biennial screening, in years (t_{r-1})	0	2	4	...	$2(r-1)$	...	2(n-1)

The probability of detecting a BC in the first exam (t_0) is

$$Det(t_0, z) = \beta(z) \int_0^z S(z-x)w(z-x)Q(x)dx \tag{11}$$

where

$$Q(t) = \int_t^\infty q(x)dx \tag{12}$$

is the tail probability of the sojourn time in the preclinical state.

The probability of detecting a BC at time t_r, $Det(t_r, z)$ takes into account if the transition to S_p was done before ($Det_{pre}(t_r, z)$) or after ($Det_{post}(t_r, z)$) the age z.

$$Det(t_r, z) = Det_{pre}(t_r, z) + Det_{post}(t_r, z) \tag{13}$$

$$Det_{pre}(t_r, z) = \beta(z+t_r) \left(\prod_{i=0}^{r-1} (1-\beta(z+t_i) \right) \int_0^z S(z-x)w(z-x)Q(t_r+x)dx \tag{14}$$

$$Det_{post}(t_r, z) = \beta(z+t_r) \sum_{j=1}^{r} \left(\prod_{i=j}^{i<r} (1-\beta(z+t_i)) \right) \int_{t_{j-1}}^{t_j} S(z+x)w(z+x)Q(t_r-x)dx \tag{15}$$

Finally, the probability of being detected in a specific disease stage, j, is obtained weighting by the stages' distribution that correspond to a specific pattern of screening π_{sc} [1,6] .

$$Det(t_r, z, j) = \pi_{sc}(t_r+z, j) Det(t_r, z) \tag{16}$$

D.1.3 BC diagnosed between two exams in the interval (t_{r-1}, t_r)

Women diagnosed in the interval (t_{r-1}, t_r) may have entered the preclinical state S_p:

- before age z
- in the interval (z, t_{r-1})

- in the interval (t_{r-1}, t_r)

We introduce an additional index, s, that allows to obtain the estimates yearly when the screening scenario has biennial periodicity or higher. Thus, $s = 0$ refers to the first year of the interval and $s = 1$ to the second year and successively.

Transition to preclinical state before age z:

$$Int_{pre}(r, z, s) = \left(\prod_{i=0}^{r-1}(1 - \beta(z + t_i))\right) \int_{t_{r-1}+s}^{t_{r-1}+s+1} \int_0^z S(z - x)w(z - x)q(\tau + x)dxd\tau \quad (17)$$

Transition to preclinical state in the interval (z, t_{r-1}):

$$Int_{post1}(r, z, s) = \sum_{j=1}^{r} \left(\prod_{i=j}^{i<r}(1 - \beta(z + t_i))\right) \int_{t_{r-1}+s}^{t_{r-1}+s+1} \int_{t_{j-1}}^{t_j} S(z + x)w(z + x)q(\tau - x)dxd\tau \quad (18)$$

Transition to preclinical stage in the interval (t_{r-1}, t_r):

$$Int_{post2}(r, z, s) = \int_{t_{r-1}+s}^{t_{r-1}+s+1} \int_{t_{r-1}}^{\tau} S(z + x)w(z + x)q(\tau - x)dxd\tau \quad (19)$$

D.1.4 BC diagnosed in an interval after the last exam n, $(t_{n-1} + s, t_{n-1} + s + 1)$.

Transition to preclinical state before age z:

$$Int_{pre}(n, z, s) = \left(\prod_{i=0}^{n-1}(1 - \beta(z + t_i)\right) \int_{t_{n-1}+s}^{t_{n-1}+s+1} \int_0^z S(z - x)w(z - x)q(\tau + x)dxd\tau \quad (20)$$

Transition to preclinical state in the interval (z, t_{n-1}):

$$Int_{post1}(n, z, s) = \sum_{j=1}^{n} \left(\prod_{i=j}^{i<r}(1 - \beta(z + t_i))\right) \int_{t_{n-1}+s}^{t_{n-1}+s+1} \int_{t_{j-1}}^{t_j} S(z + x)w(z + x)q(\tau - x)dxd\tau \quad (21)$$

Transition to preclinical state in the interval $(t_{n-1}, t_{n-1} + s + 1)$:

$$Int_{post2}(n, z, s) = \int_{t_{n-1}+s}^{t_{n-1}+s+1} \int_{t_{n-1}}^{\tau} S(z + x)w(z + x)q(\tau - x)dxd\tau \quad (22)$$

D.2 Probability of dying from BC under screening

Equations for estimating BC mortality under a screening scenario are similar to the originals from LZ's work. We used the same modifications presented in section D.1. Incidence equations integrate the product of functions S, w and q. BC mortality equations incorporate the probability density function of surviving for cases found in the screening exam, g_{ex}, or interval cases, g_{in}. Another difference between incidence and mortality equations is that in the latter the limits of the outer integral are T and $T+1$ as in Equation (6).

As in Section D.1, we introduce the index s that makes it possible to obtain the estimates yearly when the screening scenario has biennial periodicity.

D.3 Prevalence of BC under screening

Prevalence for background and screening scenarios were estimated using Equations (1) and (2) in Methods.Under-screening, $D(1,u)$ and $D(k,u)$ were estimated according to Section D.2.

As in the background scenario, we have computed $I(u)$, $D(k,u)$ and $Prev(u+k)$ for u in $40,...,79$ years of age and k in $1,...,79-u$ in a population of screened women.

E Model that estimates the false positive rates for invasive and non-invasive tests

The Cumulative False Positive Research study (RAFP) provided the false positive (FP) rates for invasive and non-invasive tests for women starting biennial exams at ages 44-45 to 68-69. The maximum number of screening rounds was six (non-published data).

We used the FP rate as the dependent variable and included the screening round and age as independent variables, in a log linear model. The model allowed us to estimate the FP rate for annual screening. We assumed that after the 6th exam the FP rate was constant. The estimated coefficients, confidence intervals and p-values were the following:

E.1 Model for FP of non-invasive tests

	Estimate	95% CI	P-value
Constant	-1.29	(-1.66, -0.92)	<0.001
x1	-0.14	(-0.17, -0.11)	<0.001
x2	-0.03	(-0.04, -0.03)	<0.001
x3	0.55	(0.42, 0.68)	<0.001

E.2 Model for FP of invasive tests

	Estimate	95% CI	P-value
Constant	-4.65	(-5.03, -4.28)	<0.001
x1	-0.15	(-0.18, -0.12)	<0.001
x2	-0.02	(-0.03, -0.01)	<0.001
x3	1.06	(0.93, 1.20)	<0.001

were:

- x1: screening round, ranges from 1 to 6
- x2: age at screening
- x3: takes value 1 if $x1 = 1$ and 0 if $x1 > 1$

References

1. Lee SJ, Zelen M: **Mortality Modeling of Early Detection Programs**. *Biometrics* 2008, **64**:386–395.

2. Rue M, Vilaprinyo E, Lee S, Martinez-Alonso M, Carles M, Marcos-Gragera R, Pla R, Espinas JA: **Effectiveness of early detection on breast cancer mortality reduction in Catalonia (Spain)**. *BMC Cancer* 2009, **9**:326.

3. Generalitat de Catalunya Departament de Sanitat i Seguretat Social Direcció General de Recursos Sanitaris: **Anàlisi de la mortalitat a Catalunya** 1983-2006, [http://www.gencat.net/salut/].

4. Instituto Nacional de Estadística: **Population data** 2007, [http://www.ine.es].

5. Institut d'Estadística de Catalunya: **Padró municipal d'habitants** 2007, [http://www.idescat.net].

6. Lee S, Huang H, Zelen M: **Early detection of disease and scheduling of screening examinations**. *Stat Methods Med Res* 2004, **13**:443–456.

7. Lee S, Zelen M: **Scheduling periodic examinations for the early detection of disease: applications to breast cancer**. *J Am Stat Assoc* 1998, **(93)**:1271–1281.

Tables

Table A.1: **Cost and effect of all screening strategies.**

Scenario	Cost ($\times 10^6$ €)	LE	YL	QALY	Cost per woman diagnosed (€)
Bg	127.3	0	0	0	25,633
B50-69	143.4	567	4,691	3,614	27,736
B50-70	144.6	590	4,812	3,722	27,781
B50-74	147.0	640	4,990	3,891	27,747
B45-69	151.5	617	5,842	4,447	29,290
B50-79	151.5	651	5,008	3,881	27,510
B45-74	153.9	666	6,038	4,633	29,279
B45-79	160.6	686	6,075	4,625	28,754
A50-69	160.8	688	6,528	5,003	30,792
B40-69	161.5	627	6,630	4,943	31,197
B40-70	162.6	649	6,751	5,051	31,220
B40-74	165.1	699	6,929	5,220	31,124
A50-74	167.0	757	6,781	5,234	31,229
B40-79	169.6	710	6,947	5,210	30,759
A50-79	175.9	769	6,800	5,199	31,046
A45-69	176.0	734	7,917	5,979	33,705
A45-74	182.1	803	8,170	6,210	34,074
A45-79	191.0	815	8,190	6,175	33,732
A40-69	195.4	768	9,117	6,756	37,250
A40-74	201.5	837	9,370	6,987	37,535
A40-79	210.4	849	9,390	6,952	37,000

Table A.2: **Sensitivity analysis for advanced care cost**. LE and QALY of dominant scenarios. The asterisk indicates the reference scenario.

**LE**					
Scenario	Baseline	$C_{ac}2$	$C_{ac}3$	$C_{ac}5$	$C_{ac}10$
Bg	$*$	$*$			
B 50-69	28,465	13,270			
B 50-70	49,184	20,773			
B 50-74	50,188	21,774	$*$	$*$	
A 50-74	170,304	141,892	113,480	56,657	$*$
A 45-74	330,098	301,686	273,273	216,448	74,385
A 40-74	573,062	544,649	516,236	459,410	317,345
A 40-79	715,941	687,519	659,097	602,254	460,145

**QALY**					
Scenario	Baseline	$C_{ac}2$	$C_{ac}3$	$C_{ac}5$	$C_{ac}10$
Bg	$*$	$*$			
B 50-69	4,469	2,084			
B 50-70		4,375			
B 50-74		6,369	$*$	$*$	
B 45-69	9,693				
B 45-74	12,633	8,182	7,166		
A 45-69	16,411	14,977			
A 40-69	24,975				
A 50-74				4,945	$*$
A 45-74		18,295	13,003	10,162	3,492
A 40-74	26,720	23,737	22,499	20,022	13,831

Table A.3: **Sensitivity analysis for LE.** LE of dominant scenarios when the follow-up cost is 2- or 10-fold ($C_{fw}2$ and $C_{fw}10$), the initial treatment is 2- or 10-fold ($C_{it}2$ or $C_{it}10$), the follow-up time is 11 or 21 years ($T_{fw}11$ and $T_{fw}21$), the ratio of non invasive test Screening/background is 1 or 3 (R1 and R3), the 50% of the population is screened $Sc(0.5)$, and the price of invasive test for screened women is doubled (B2). The asterisk indicates the reference scenario.

Scenario	Baseline	$C_{fw}2$	$C_{fw}10$	$C_{it}2$	$C_{it}10$	$T_{fw}11$	$T_{fw}21$	R1	R3	Sc(0.5)	B2
Bg	*	*	*	*	*	*	*	*	*	*	*
B 50-69	28,465	31,215	53,217	30,203	44,110	30,978	34,138	23,114	30,248	48,450	31,369
B 50-70	49,184	58,325	131,456	57,478				43,691	51,015		54,386
B 50-74	50,188	62,746	163,206	67,189		54,360	54,360	44,694	52,020	93,408	54,839
A 50-69					88,107						
A 50-74	170,304	173,769	201,488	162,508	203,702	174,232	178,820	170,257	170,319	287,543	174,872
A 45-74	330,098	332,787	354,296	321,468	252,426	332,835	336,638	314,431	335,321	458,095	341,279
A 40-74	573,062	578,011	617,608	573,806	579,756	577,101	583,270	554,996	579,084	747,445	588,311
A 40-79	715,941	765,795	1,164,622	970,210	3,004,363	715,941	715,941	699,920	721,281	983,578	744,524

Table A.4: **Sensitivity analysis for QALY.** QALYs of dominant scenarios. The asterisk indicates the reference scenario. See legend in Table A.3.

Strategy	Baseline	$C_{fw}2$	$C_{fw}10$	$C_{it}2$	$C_{it}10$	$T_{fw}11$	$T_{fw}21$	$R1$	$R3$	$Sc(0.5)$	B2
Bg	*	*	*	*	*	*	*	*	*	*	*
B 50-69	4,469	4,901	8,356	4,742	6,926	4,864	5,360	3,629	4,749	7,615	4,925
A 50-69					7,669						
B 45-69	9,693	9,897	11,528	9,476		9,931	10,035	8,777	9,999	13,923	10,201
B 45-74	12,633	15,530				13,876	13,876	10,911	13,207	23,606	13,880
B 40-70										24,450	
A 45-69	16,411	16,301	18,248	15,324	11,851	16,528	16,993	16,644	16,334	25,351	16,683
A 40-69	24,975	25,191	26,917	25,008	25,267	25,151	25,420	24,188	25,238	32,749	25,640
A 40-74	26,720	30,173	57,795	30,463	60,405	28,119	28,119	25,212	27,223	49,065	28,346
